# Supplementary material for: Pollen from multiple sunflower cultivars and species reduces a common bumblebee gut pathogen
Source: R Soc Open Sci. 2019 Apr 3;6(4):190279. doi: 10.1098/rsos.190279 (PMC6502360; doi:10.1098/rsos.190279)
Supplement: Supplemental Methods [file rsos190279supp1.docx]

**Pollen from multiple sunflower cultivars and species reduces a common bumble bee gut pathogen**

George M. LoCascio^1*^, Luis Aguirre^2^, Rebecca Irwin^3^, and Lynn S. Adler^2^

^1^ Department of Environmental Conservation, University of Massachusetts, Amherst, MA 01003, USA

^2^ Department of Biology, University of Massachusetts, Amherst, MA 01003, USA

^3^ Department of Applied Ecology, North Carolina State University, Raleigh, NC 27695, USA

^*^ corresponding author: glocascio@umass.edu, ph 617-372-6384, fax 413-545-3243

**Supplemental methods**

*Plant propagation information*

All seeds were sown in SUNGRO Horticulture medium (SunGro Horticulture Canada Ltd., Seba Beach, AB TOE 280, Canada) in 50-plug trays with natural lighting. They were germinated at 21˚C and misted every 15 minutes for 30 seconds from sunrise to sunset on a mist top bench. When 50% of the seedlings in a tray reached the four true-leaf stage, the tray was relocated to a different greenhouse with natural light plus 14 hours of artificial supplementary lighting consisting of 50% 400W high pressure sodium and 50% 400W metal halide lights. Plants were watered as needed. The plants ranged from 18-60 cm in height when transplanted to the field site. *H. petiolaris* was transplanted into 15-cm pots and remained in the greenhouse due to small sample sizes. *H. petiolaris* was fertilized twice, on April 20 and May 13, 2016, and *H. argophyllus* once on May 13, 2016 with Peters Professional 20-10-20 Peat lite (JR Peters Inc. Allentown PA, USA) at 350 ppm. Other taxa were not fertilized. *Helianthus annuus* ‘Dwarf’ cultivar was sown directly into soil in the field. Pollen collection methods are located in the main body of the methods section of the manuscript.

*Seed scarification protocol*

Seeds were sterilized with a 2% bleach (The Chlorox Co, Oakland CA, USA) and 1% Triton-X 100 (Sigma-Aldrich, St. Louis Missouri, USA) solution for 10 minutes, stirring for 10 seconds every minute. Afterwards, they were rinsed in distilled water for 5 minutes, stirring for 10 seconds every minute. After rinsing, a small section of the blunt end of each achene was removed with scissors. The seeds were then placed on moist filter paper in 95 mm petri dishes, sealed with parafilm and placed in an incubator for 48 hours at 27˚ C. After 48 hours, the remaining seed coat was removed by hand and cotyledons were placed on a new petri dish with new moist filter paper in an incubator for an additional 24-48 hours. When the radicle grew to > 10 mm on ~50% of the seeds, petri dishes were moved to a lab bench in ambient light for 3 days, after which they were transplanted into the soil growing medium and grown in the greenhouse as described above in ‘*Plant propagation information.*’

*Pollen collection and preparation*

We collected pollen by hand (i.e., granular pollen without nectar or anther tissue) and with honey bees and brought it to the lab daily for storage at -20˚C until trials began. For most taxa, we were only able to collect by hand by honey bee due to time constraints. However, for three *H. annuus* taxa (‘Black Oil Seed’, ‘Germany’, and ‘wild California’), we collected by both methods, allowing direct comparisons between collection methods within taxa. Hand-collected pollen was collected with paint brushes into aluminum foil envelopes and then stored in a freezer inside 5 mL plastic vials. Honey bee-collected pollen was collected using pollen traps (Mann Lake Ltd. Hackensack, Minnesota, USA) in honey bee hives from the UMass-Amherst Apiary. Traps were checked and pollen was collected weekly from July to October 2016. The pollen from all the wild *H. annuus* and the ‘Dwarf’ cultivar were honey bee-collected inside 3.6 m x 9.7 m x 3m tents (Figure S1A, B) made of polyester fabric (Osgood Textile, West Springfield, Massachusetts, USA) and fine mesh cloth (Phifer Incorporated, AL, USA) with a metal frame (Delta Canopies, McKinney, TX, USA), which excluded other pollinators. Due to logistics, we did not use tents for all taxa grown at the field station. *Solidago* spp., *H. annuus* ‘Cobalt II’, and *H. annuus* ‘Black Oil Seed’ were present at natural sites or commercial farms, and were honey bee-collected without tents. Pollen collected from *Solidago* spp. and *H. annuus* ‘Cobalt II’ sites also contained pollen from other species. After microscopic examination, we sorted and excluded pollen from other species, and separated the yellow and orange pollen from our taxa in case colors indicated phytochemical differences that could affect *C. bombi* infection. We also note that *H. annuus* ‘White’ had white pollen, while all other taxa had yellow or orange pollen.

*Tent assembly*

  The metal tent frame (Delta Canopies, McKinney, Texas, USA) was assembled according to manufacturer instructions with slight modifications. We removed 12, 0.75m joint pieces, which reduced the width of the tent from 4.8m to 3.6m; length was 9.7 m and height was 3 m.  The 9 joints where the legs and the roof of the frame connected were reinforced by 0.6m metal conduit pieces. The conduits were attached to roof sections spanning the joints perpendicular to the vertical legs with six to eight zip ties. The frame was also reinforced with three evenly spaced ratchet straps pulled taught, spanning the width of the frame parallel to the ground. Next, the polyester fabric and mesh was draped evenly over the frame and held in place with 10 2.54 cm PVC snap clips (Johnny’s Select Seeds, Winslow, Maine, USA), one per leg. Four pieces of 0.6m factory-provided angle iron, two on each side near the tent ends, were hammered into the ground and reinforced by 2 0.6m pieces of 7.62cm rebar to provide an anchor for rope that spanned the top of the tent to secure the frame and fabric in place. Finally, a trench approximately 12cm deep and 6cm wide was dug around the perimeter of the tent, except for a space of 2m across the one of the narrow sides, which served as an entrance. Excess fabric was rolled around lumber (4cm x 8cm, various lengths) and buried in the trenches. To secure the entrance, fabric was rolled on one piece of lumber and weighted down with concrete bricks and soil.

**Table S1.** Description, source, propagation methods and sample size for taxa used to collect experimental pollen. ‘Taxa’ refers to the plant species or cultivar used in the experiment. ‘Pollen color’ is usually yellow or orange, but sometimes white. ‘Sample size’ refers to the number of bees that went through the experiment with that pollen treatment. ‘Collection type’ is bee, hand, or both, indicating how the pollen was collected. ‘Bee’ was collected with honey bees using pollen traps, ‘hand’ was collected by hand using paint brushes and colleting the pollen into aluminum foil envelopes, and ‘both’ was when we used both methods. ‘Source’ refers to where we obtained the pollen from, and the GRIN seeds are accompanied with the plot ID number (PI) that the seeds were obtained from. In ‘location,’ ‘UMass’ refers to the Crop and Animal Research and Education Center in South Deerfield, Massachusetts (42°28'39.6"N 72°34'51.1"W), operated by the University of Massachusetts, Amherst. ‘Seed scarification,’ ‘Transferred to 15-cm pot,’ and ‘Date transferred to field’ refer to propagation methods for taxa we grew. All dates are from 2016. ‘Amount of plants’ refers to the total acres or the total number of plants that were grown.

| **Taxa** | **Pollen color** | **Sample size**  **(n)** | **Collection type** | **Source** | **Location** | **Seed Scarification** | **Transferred to 15-cm pot** | **Date Transferred to field** | **Amount of plants** |
| --- | --- | --- | --- | --- | --- | --- | --- | --- | --- |
| *Solidago* spp | Yellow | 36 | Bee | East Leverett Meadow | Massachusetts  42˚43’91.80” N, -72˚48’68.03” W | N/A | N/A | N/A | ~5 acres |
| *Solidago* spp | Orange | 48 | Bee | East Leverett Meadow | Massachusetts  42˚43’91.80” N, -72˚48’68.03” W | N/A | N/A | N/A | ~5 acres |
| *H. annuus*,  Black Oil Seed | Yellowish orange | Bee=51  Hand=42 | Both | Laurenitis Farm | Massachusetts  42˚44’17.77” N, -72˚55’04.64” W | N/A | N/A | N/A | ~1 acres |
| *H. annuus*,  Cobalt II | Yellow | 41 | Bee | Messa Farm | Wisconsin  44˚73’16.91” N, -91˚94’86.32” W | N/A | N/A | N/A | ~75 acres |
| *H. annuus*,  Cobalt II | Orange | 47 | Bee | Messa Farm | Wisconsin  44˚73’16.91” N, -91˚94’86.32” W | N/A | N/A | N/A | ~75 acres |
| *H. annuus*, China | Orange | 49 | Bee | Changge Hauding Wax Industry | China | N/A | N/A | N/A | Unknown |
| Buckwheat | Brown | 57 | Bee | Changge Hauding Wax Industry | China | N/A | N/A | N/A | Unknown |
| *H. annuus*, Germany | Yellowish orange | Bee=14  Hand=11 | Both | GRIN Seeds  Pl-650375 | UMass | No | No | June 17 | ~300 plants |
| *H. annuus*, wild  California | Yellowish orange | Bee=15  Hand=41 | Both | GRIN Seeds  Pl-613732, Pl-649815, Pl-649816 | UMass | Yes | No | July 12 | ~300 plants |
| *H. annuus*,  ‘Dwarf’ | Yellowish orange | 36 | Bee | The Chas. C. Hart Seed Co  Wethersfield, CT, USA | UMass | No | No | July 8  (directly sown) | ~300 plants |
| *Helianthus argophyllus* | Yellowish orange | 14 | Hand | GRIN Seeds  Pl-435630, Pl-494569 | UMass | Yes | Yes | June 17 | ~300 plants |
| *H. annuus*, wild Texas | Yellowish orange | 35 | Hand | GRIN Seeds  Pl-613728, Pl-649810, Pl-649811 | UMass | Yes | No | June 17 | ~300 plants |
| *H. annuus*, wild North Dakota | Yellowish orange | 35 | Hand | GRIN Seeds  Pl-613724, Pl-613725, Pl-613750 | UMass | Yes | No | June 27 | ~300 plants |
| *H. annuus*,  white | White | 18 | Hand | GRIN Seeds  Pl-650655 | UMass | Yes | No | June 17 | ~300 plants |
| *Helianthus petiolaris* | Yellowish orange | 7 | Hand | GRIN Seeds  Pl-435825, Pl-435826, Pl-435827 | UMass | Yes | Yes | N/A | ~30 plants |
| Wildflower mix | Various | 53 | Bee | Koppert Biological Systems | Minnesota | N/A | N/A | N/A | Unknown |

**Figure S1.** (A) Outside and (B) inside of tents used to house honey bee hives for pollen collection or to exclude other pollinators when hand-collecting pollen. (C, D) Individual bee isolation deli cup set up. (C) Left deli cup housed the bee and then was inserted into the right deli cup, where (D) the bee could have access through the mesh bottom to 10 ml of sugar solution via cotton wick inserted into a 95 mm petri dish with a hole cut in the top to allow the wick. Also shown is pollen in a “pollen cup,” which was the cap of a 1.5 mL microcentifuge tube.
