# Supplementary material for: Pollen from multiple sunflower cultivars and species reduces a common bumblebee gut pathogen
Source: R Soc Open Sci. 2019 Apr 3;6(4):190279. doi: 10.1098/rsos.190279 (PMC6502360; doi:10.1098/rsos.190279)
Supplement: README_CSV [file rsos190279supp5.rtf]

This file describes the CSV files associated with the paper “Pollen from multiple sunflower cultivars and species reduces a common bumble bee gut pathogen”George M. LoCascio, Luis Aguirre, Rebecca Irwin, and Lynn S. Adler(I) CSV files for 19 pollen treatments~~~~~~~~~~~~~~~~~~~~~~~~~~~~~~~~~~~~Sflower1.csvBeeID: A unique identifier for each experimental bee.Colony: An arbitrary name for the uninfected parent colony from which each experimental bee came.Treatment: Refer to Table S1 of Appendix S1 for exact species, cultivar, wild type or relative.Inoc.date: Month/Day/Year in which bee was entered into the experimentDissect Date: Month/Day/Year in which bee was dissected; should be 7 days after InocDate. Count: The number of Crithdia cells counted in 0.02 µl of gut solution. Count ID: Initials of person who counted the gut samplesNotes: anything usual when counting or dissecting experimental beesWings: The length of radial cell of the right forewing, measured in mm, which calculated using the relationship Wing_length_mm = OcularUnits *(13/20) or Wing_length_mm = OcularUnits *(14/20), depending on the calibration for that slide. Vortex time: Time gut sample was mixed Count time: time that gut sample was countedDissect ID: identifying initials of person dissecting the experimental beesCount day of week: day of the week bees exited the experiment(I) CSV files for 19 pollen treatments and time to death before the end of the experiment~~~~~~~~~~~~~~~~~~~~~~~~~~~~~~~~~~~~Death_by_flower.csvBeeID: A unique identifier for each experimental bee.Treatment: Refer to Table S1 of Appendix S1 for exact species, cultivar, wild type or relative.Inoc.date: Month/Day/Year in which bee was entered into the experimentCount day of week: day of the week bees exited the experimentDissect Date: Month/Day/Year in which bee was dissected; should be 7 days after InocDate. Dissect ID: identifying initials of person dissecting the experimental beesVortex time: Time gut sample was mixed Count time: time that gut sample was countedCount: The number of Crithdia cells counted in 0.02 µl of gut solution. Count ID: Initials of person who counted the gut samplesDeath date: the date Day/Month/Year experimental bees died before leaving the experimentColony: An arbitrary name for the uninfected parent colony from which each experimental bee came.Wings: The length of radial cell of the right forewing, measured in mm, which calculated using the relationship Wing_length_mm = OcularUnits *(13/20) or Wing_length_mm = OcularUnits *(14/20), depending on the calibration for that slide. Time.to.death: numerical value representing the amount of days it took for the experimental bees to die. These bees died before the end of the experiment.Death.before.dissect: Binary if a bee died or not before the end of the experiment. O is that the bee did not die before the end of the experiment and the bee successfully made it through to the end of the experiment. 1 is that the bee died before the end of the experiment.
