# Supplementary material for: Pollen from multiple sunflower cultivars and species reduces a common bumblebee gut pathogen
Source: R Soc Open Sci. 2019 Apr 3;6(4):190279. doi: 10.1098/rsos.190279 (PMC6502360; doi:10.1098/rsos.190279)
Supplement: README.R_Scripts [file rsos190279supp6.rtf]

This file describes the R script files associated with the paper “Pollen from multiple sunflower cultivars and species reduces a common bumble bee gut pathogen”George M. LoCascio, Luis Aguirre, Rebecca Irwin, and Lynn S. AdlerThe supplemental materials  include CSV files from which the R scripts read in data to be analyzed.  These CSV files were extracted from original data files in XLS or XLSX format. The CSV files are explained in the README_CSV.txt file. The CSV files are included in the supplemental materials so that the R scripts will run "out of the box" to replicate analyses in the paper. (I) General comments on the R scripts Many of the R scripts include a "set the working directory" block at the beginning, which you will need to modify to your own directory.   (II) Script files for the trials across 19 pollen treatmentsSunflower experiment script (full).RThis scripted uses the CSV files Sflower1.csv and Death_by_Sflower.csv which must be read in to the working directory.This script fits the negative binomial regression models for pathogen counts reported in the manuscript, and then does the simulation-based goodness of fit tests for the negative binomial model for each treatment.  This script does the screening of covariates for consideration in the regression models for pathogen counts. This script makes several of the figures for the manuscript and online SI.It also fits the time to death of bees that did not make it to the end of the experiment and died early.This script also compares the direct comparison and pooled comparison of honey bee collected pollen and hand collected pollen and how they suppressed Crithidia or not.This scripted also compares how the individual species of sunflower and Asteraceae relatives when grouped suppressed Crithidia or not.
